# Skin and soft-tissue infections in suburban primary care: epidemiology of methicillin-resistant *Staphylococcus aureus *and observations on abscess management

**DOI:** 10.1186/1756-0500-4-33

**Published:** 2011-02-07

**Authors:** Charles Weiss, Peggy Kaminsky, John Boggs, Catherine Ley

**Affiliations:** 1 Urgent Care Palo Alto Medical Foundation 795 El Camino Real Palo Alto, CA 94301, USA; 2 Infection Control Palo Alto Medical Foundation 795 El Camino Real Palo Alto, CA 94301, USA; 3 Infectious Diseases, Internal Medicine Palo Alto Medical Foundation 795 El Camino Real Palo Alto, CA 94301, USA; 4 Caradon Consulting 338 Oakview Dr. San Carlos, CA 94070, USA

## Abstract

**Background:**

Reports from urban medical centers suggest that methicillin-resistant *Staphylococcus aureus *(MRSA) has become the most common cause of skin and soft-tissue infections (SSTIs). Risk factors for MRSA have been identified but have not been clinically useful.

**Findings:**

From May 2006-April 2007, we performed an observational study of 529 SSTIs among ambulatory patients in the urgent care departments of a large suburban primary-care practice. SSTIs were included if they produced pus or fluid. The proportion of MRSA was determined overall (defined as prevalence) and by SSTI diagnosis. Potential risk factors for MRSA were examined with multivariate analysis, and descriptive statistics were generated for follow-up and abscess management. The prevalence of MRSA was 22% and did not rise during the study. MRSA was isolated from 36% of abscesses, 15% of cellulitis, and 14% of other SSTIs. Independent risk factors for MRSA included a prior history of MRSA (adjusted odds ratio [aOR], 41.05; 95% confidence interval [CI], 11.4-147.3), a close contact with prior MRSA (aOR, 12.83; 95% CI, 4.2-39.2), erythema ≥10 cm (aOR, 2.59; 95% CI, 1.5-4.4), and abscess diagnosis (aOR, 3.19; 95% CI, 2.1-5.0). Prior MRSA had a positive predictive value of 88% for current MRSA. When both abscess diagnosis and erythema ≥10 cm were present, the proportion of MRSA was 59%. The vast majority of SSTIs (96 percent) resolved or improved within one week. Most abscesses, even small ones, were treated with antibiotics. Resource utilization was highest in those abscesses with erythema ≥10 cm.

**Conclusions:**

The prevalence of MRSA is relatively low among SSTIs in suburban primary care. However, MRSA is common in the subgroup of abscesses with large erythema. While the effectiveness of adjunctive antibiotic therapy for large abscesses is unknown, drugs chosen for these infections should be active against MRSA. Most non-abscess SSTIs do not require treatment with a MRSA-active drug, and antibiotics are probably overused for small abscesses. A history of prior MRSA should be recorded in a patient's health record.

## Background

The prevalence of methicillin-resistant *Staphylococcus aureus *(MRSA) in skin and soft-tissue infections (SSTIs) has reached epidemic proportions in the United States (US) according to studies published since 2004 [1-7]. The emergence of MRSA has been particularly rapid in some areas [3,4,7]. MRSA is resistant to beta-lactam antibiotics such as cephalexin and dicloxacillin which are traditionally used to treat SSTIs [8]. With the rising prevalence of MRSA, many SSTIs may be inadequately treated if clinicians continue to rely on beta-lactam agents. Conversely, SSTIs not caused by MRSA may be treated unnecessarily with MRSA-active drugs.

Most MRSA isolates from SSTIs in the United States are descended from a clone of MRSA known as USA-300 [5,6]. These isolates contain the SCC*mec *IV gene complex which conveys methicillin resistance [2,9], and are termed community-associated MRSA (CA-MRSA) for lack of traditional risk factors linked to healthcare-associated infection [8,9]. While CA-MRSA isolates are usually susceptible to tetracycline and trimethoprim-sulfamethoxazole (TMP/SMX), and have been universally susceptible to vancomycin, substantial resistance to both clindamycin and fluoroquinolones has been reported [2,4,6,7]. Multiple risk factors for CA-MRSA have been identified in both outbreak [10-12] and endemic [2,5,6,13-16] settings, but they appear to have limited clinical usefulness [6,14]. Follow-up data suggest timely resolution in most SSTIs caused by MRSA [6,17-20]. However, there have been reports of delayed treatment response [21-24], high hospitalization rates [4,25], and severe infections [26,27].

Early studies of endemic CA-MRSA were performed primarily in outpatient clinics, emergency departments, and hospitals associated with urban medical centers that serve indigent populations [1-7]. In addition, the types and sizes of SSTIs examined in some of these studies were not clearly defined [1,3-5,7]. To address these limitations, we investigated the epidemiology of MRSA in SSTIs among ambulatory patients from suburban areas in the San Francisco Bay Area (Bay Area). We classified SSTIs by diagnosis group and maximum erythema size, and also described initial treatment decisions and resource utilization in the management of abscesses.

## Methods

This was an observational study of purulent or fluid-producing SSTIs that were sampled for bacterial culture among ambulatory patients from May 1, 2006 through April 30, 2007. Incision and drainage (I&D) of abscesses and culturing of pus and fluid from SSTIs were usual care prior to the study. Clinical decisions regarding procedures, cultures, antibiotics, and charting in the electronic health record (EHR) remained at the discretion of providers.

The study took place in the adult and pediatric urgent care departments (urgent care) of the Palo Alto Medical Foundation, Palo Alto Division (PAMF-PA), which have over 50,000 patient visits annually. PAMF-PA provides primary and specialty care to an ambulatory patient population of approximately 300,000 persons in the Bay Area, The primary care providers (PCPs) in the family medicine, internal medicine, and pediatrics departments at PAMF-PA frequently refer patients to urgent care for I&D of abscesses and for intravenous infusions of antibiotics. The study was funded by PAMF-PA.

Patients of any age were considered for inclusion in the study if a new SSTI was diagnosed by an urgent care provider and a bacterial culture was obtained from the infection site. An SSTI was considered new if there was no previous history of SSTI, or if greater than one month had elapsed since resolution of erythema and pus or fluid in a previous SSTI. Patients were excluded if they had a nonbacterial skin disorder (e.g. herpes simplex infection or trauma without infection) or a noncutaneous bacterial infection (e.g. vaginitis or peri-rectal abscess). Patients were not excluded if they had a history of risk factors for healthcare-associated MRSA.

The proportions of MRSA, methicillin-sensitive *Staphylococcus aureus *(MSSA), and Streptococcal species were determined overall and by three diagnosis groups: abscesses (discrete collections of pus including furuncles and carbuncles), cellulitis (spreading erythema with bullous or open purulent, moist, or fluid-producing areas), and other SSTIs (felon, folliculitis, impetigo, paronychia, septic bursitis, inflamed sebaceous cyst, and miscellaneous). The overall proportion of a bacterial isolate was termed its prevalence. Demographics and potential risk factors for MRSA versus non-MRSA culture results were examined. Erythema, an easily ascertained measure of infection size, was defined as the maximum dimension of erythema in any direction measured in cm. If erythema was not recorded in the EHR, an estimate was imputed using standard anthropometric tables [28] where possible. Erythema ≥10 cm was considered "large" erythema. Injection drug use was not examined because a previous case series of MRSA SSTIs at our institution revealed no patient with this history (unpublished data). Follow-up status was assessed as better or resolved, worse or no change, or lost to follow-up at last patient contact within seven days of the initial visit. Initial treatment selections for abscesses included antibiotic therapy (started or continued at the initial visit), I&D, both antibiotic and I&D, or neither antibiotic nor I&D. An antibiotic selection was considered "active" if the isolate was confirmed susceptible to the drug by laboratory testing. Repeat I&D and ≥3 follow-up visits were considered variables potentially affected by initial antibiotic selections. Resources utilized for abscess management (in addition to an oral antibiotic and/or initial I&D) were outpatient intravenous therapy (IV), hospitalization, repeat I&D, and ≥3 follow-up visits. Resource utilization was described by erythema size.

Reports listing all wound and body fluid culture results were reviewed thrice weekly to identify possible SSTIs for the study. Inclusion of SSTIs and assignment of diagnosis group was based on initial provider assessment and consensus between two authors (CW and PK). The visit during which the initial bacterial culture was performed was the initial visit. Specimens for aerobic culture were obtained with a Culturette EZ II™ transport swab (Becton Dickinson, Franklin Lakes, New Jersey) and were processed and cultured at the Mills-Peninsula Hospital Laboratory (San Mateo, CA) utilizing standard techniques [29]. The Dade Behring Microscan system was used to determine antibiotic susceptibilities. The D-zone test was done on isolates that were erythromycin-resistant and clindamycin-susceptible to confirm clindamycin susceptibility.

A standardized SSTI template was available for provider use in the EHR. Data were extracted from the SSTI template or other EHR notes. As a proxy for individual income and education data, the socioeconomic status (SES) of the zip codes in which patients resided was examined using the US Postal Service [30] and US Census 2000 [31]. Follow-up status was determined by EHR review and telephone calls made on days 3 and 7 after the initial visit. Up to two calls per patient were made on both days. Initial treatment selections and resource utilization for abscesses were recorded. The total number of I&D procedures performed in urgent care during the study period was provided by the information technology department at PAMF-PA.

All SSTIs, including those with no growth, were included in the analysis. Descriptive statistics were generated after data entry in Epi Info software, version 3.3.2, February 9, 2005 (CDC, Atlanta, Georgia). Analysis of potential risk factors for MRSA was performed for MRSA versus non-MRSA and for MRSA versus MSSA. Continuous variables were analyzed with the t-test and dichotomous variables with the chi-square test. Multivariate analysis was performed using logistic regression with variables either considered clinically important or with a significant association to specific outcome variables at the 0.05 significance level, using SAS, version 8.2 (Cary, North Carolina). Odds ratios (OR) and 95 percent confidence intervals (CI) were calculated. Separate models were created for demographical/historical variables and clinical variables.

The Institutional Review Board of the PAMF-PA approved the study. Verbal consent was obtained for follow-up telephone calls.

## Results

**Figure 1:**
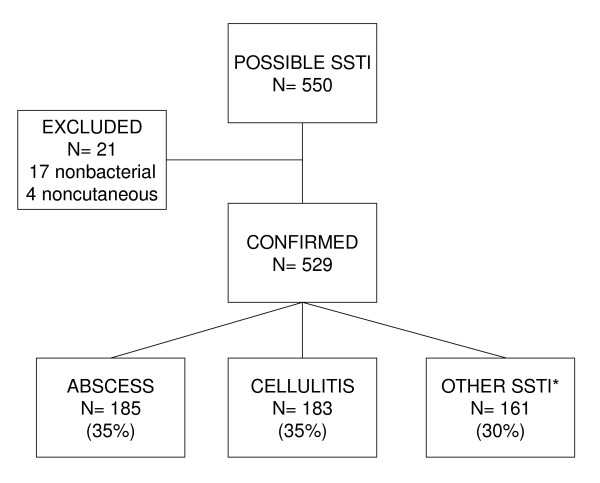
**Distribution of skin and soft-tissue infections within the study**. Abbreviations: SSTI, skin and soft-tissue infection. *Felon (5), folliculitis (20), impetigo (25), finger paronychia (45), toe paronychia (14), olecranon bursitis (14), prepatellar bursitis (3), sebaceous cyst (32), miscellaneous (3).

There were 513 patients (Table 1) with 529 confirmed SSTIs (Figure 1) during the 12 months of the study. Zip codes were available for 502 patients, 468 (93 per cent) of whom resided in the Bay Area. Of the Bay Area patients, 428 (91 percent) had addresses outside the major cities of San Francisco, San Jose, and Oakland. The vast majority of Bay Area patients lived in zip codes with incomes and educational levels above the national median. Nearly 60 percent of patients had a designated PCP at PAMF-PA. SSTIs were divided almost equally among abscesses, cellulitis, and other SSTIs (Figure 1). During the study, 362 I&D procedures were performed for SSTIs in urgent care. Of these, 264 (73 percent) had a bacterial culture, and 245 (68 percent) were included in the study.

**Table 1:** Demographical characteristics (N = 513)

Characteristic	No. (%)
Age (years)	
Median	41
Range	17 days-93 years
0-17	105 (20)
18-64	313 (61)
≥65	95 (19)
Male sex	287 (56)
Primary care at PAMF-PA	298 (58)
High income zip codes*^(1)^	463 (99)
High education zip codes*^(2)^	459 (98)

Abbreviation: PAMF-PA, Palo Alto Medical Foundation, Palo Alto division.

* Based on 468 Bay Area residents, proportion in zip code of (1) households with median household income > national median

household income; (2) residents aged ≥25 yrs with a bachelor's degree or higher greater than the proportion nationwide.

(Definitions from the U.S.Census.)

Ninety percent of cultures were positive for at least one bacterial isolate. The prevalence of MRSA was 22 percent, lower than that of MSSA but higher than Streptococcal species (Table 2). MRSA accounted for only 42 percent of *S. aureus *isolates. No culture was positive for both MRSA and MSSA, while one culture grew MRSA and a Streptococcal species. Other organisms in the non-MRSA group that were isolated more than once included coagulase-negative *Staphylococci *(100), *E. coli *(13), diphtheroids (8), *Pseudomonas *spp. (8), *Enterobacter *spp. (5), *Peptostreptococcus *spp. (5), *S. lugdunensis *(4), *H. influenzae *(3), *Proteus mirabilis *(3), *Serratia *spp. (3), *Bacteroides *spp. (2), *Pasturella multocida *(2), and *Propionibacterium *spp. (2).

**Table 2:** Proportion of bacterial isolates in skin and soft-tissue infections overall and by diagnosis group

Diagnosis group	MRSA, No. (%)	MSSA, No. (%)	Strep, No. (%)
All SSTI (N = 529)	115 (22)	161(30)	69 (13)
Abscess (N = 185)	66 (36)	49 (26)	19 (10)
Cellulitis (N = 183)	27 (15)	58 (32)	25 (14)
Other SSTIs (N = 161)	22 (14)	54 (34)	25 (16)

Abbreviations: MRSA, methicillin-resistant *Staphylococcus aureus*; MSSA, methicillin-sensitive *Staphylococcus aureus*; Strep, Streptococcal species; SSTIs, skin and soft-tissue infections.

The proportion of MRSA was highest in abscesses and lower in cellulitis and other SSTIs. If cases of folliculitis (10 of 20 [50 percent] positive for MRSA) were excluded from the other SSTIs group, the proportion of MRSA was only 8 percent. The monthly prevalence of MRSA and the monthly frequency of all SSTIs showed no overall increase during the study period.

Cumulative susceptibilities for MRSA isolates were 100 percent to TMP/SMX and vancomycin, 94 percent to tetracycline, 88 percent to clindamycin, and 45 percent to levofloxacin. Among the 14 MRSA isolates resistant to clindamycin, five were identified as having inducible resistance by the D-zone test.

Independent demographical and historical risk factors for MRSA versus non-MRSA were age <65 years, a reported insect bite, a prior history of MRSA, a close contact with a history of MRSA, and a current antibiotic (in all cases a drug started previously for the SSTI) (Table 3). Prior MRSA had a positive predictive value for current MRSA of 88 percent. Admission to a hospital, nursing home, or dialysis unit within the last 12 months was rare regardless of culture result. Independent clinical risk factors for MRSA were erythema ≥10 cm (80 of 500 [16 percent] erythema measurements were imputed) and abscess diagnosis (Table 4). The proportion of MRSA varied by erythema size in abscesses and was highest (59 percent) in those with ≥10 cm erythema (Table 5). The same risk factors for MRSA were identified in an analysis of MRSA versus MSSA (results not shown) aside from age <65. Providers used the EHR SSTI template to record historical and clinical findings in 280 of 529 SSTI (53 percent). Template use was not associated with MRSA (p = 0.10).

**Table 3:** Demographical and historical variables: overall, and MRSA vs. non-MRSA

	All isolates, No. (%)	MRSA, No. (%)	Non-MRSA, No. (%)	Unadjusted OR (95% CI)	Adjusted OR (95% CI)^*^
	(N = 529)	(N = 115)	(N = 414)		
Age < 65 years	432 (82)	104 (90)	328 (79)	2.47 (1.3-4.8)	2.25 (1.0-4.9), *P *= 0.002
Male sex	296 (56)	67 (58)	229 (55)	1.12 (0.7-1.7)	-
Itching†	92 (17)	29 (25)	63 (15)	1.88 (1.1-3.1)	-
Trauma†	167 (32)	24 (21)	143 (35)	0.50 (0.3-0.8)	-
Surgery†	37 (7)	5 (4)	32 (8)	0.54 (0.2-1.4)	-
Insect bite	75 (14)	33 (29)	42 (10)	3.56 (2.1-6.0)	4.18 (2.4-7.4), *P *< 0.001
Prior MRSA	26 (5)	23 (20)	3 (1)	34.3 (10.1-116.5)	41.05 (11.4-147.3), *P *< 0.001
Contact MRSA‡	22 (4)	17 (15)	5 (1)	14.18 (5.1-39.4)	12.83 (4.2-39.2), *P *< 0.001
Allergic rhinitis	79 (15)	19 (17)	60 (15)	1.17 (0.7-2.0)	-
Eczema	48 (9)	9 (8)	39 (9)	0.82 (0.4-1.7)	-
Diabetes	40 (8)	10 (9)	30 (7)	1.22 (0.6-2.6)	-
Current antibiotic§	112 (21)	31 (27)	81 (20)	1.52 (0.9-2.4)	2.21 (1.3-3.8), *P *= 0.05
Hospitalization¶	41 (8)	9 (8)	32 (8)	1.01 (0.5-2.2)	-

Abbreviations: MRSA, methicillin-resistant *Staphylococcus aureus*; OR, odds ratio; CI, confidence interval.

*Adjusted for age <65, insect bite, previous MRSA, contact with MRSA patient, and current antibiotic use.

†A history of acute itching, trauma, recent surgery, or possible insect bite refers to the infected area.

‡A person with a prior history of MRSA with whom the patient had close contact (e.g. a household member).

§Current antibiotic refers to an antibiotic the patient was already taking at study entry.

¶Admission to hospital, skilled nursing facility, or dialysis unit in the last 12 months.

**Table 4:** Clinical variables: overall, and MRSA vs. non-MRSA

	All isolates, No. (%) of observations	MRSA, No. (%)	Non-MRSA, No. (%)	Unadjusted OR (95% CI)	Adjusted OR (95% CI)*
Temperature, ≥37.8°C	30/511 (6)	7/112 (6)	23/399 (6)	1.09 (0.5-2.6)	-
Erythema ≥10 cm	93/500 (19)	38/110 (35)	55/390 (14)	3.2 (2.0 - 5.2)	2.59 (1.5-4.4), *P *< 0.001
Location	(N = 529)	(N = 115)	(N = 414)		
Trunk†	180 (34)	54 (47)	126 (30)	2.01 (1.3-3.1)	-
Head and neck	70 (13)	12 (10)	58 (14)	0.97 (0.5-1.9)	-
Extremities	279 (53)	49 (43)	230 (56)	1	-
Diagnosis group	(N = 529)	(N = 115)	(N = 414)		
Abscess	185 (35)	66 (57)	119 (29)	3.33 (2.2-5.1)	3.19 (2.1-5.0), *P *< 0.001
Cellulitis	183 (35)	27 (23)	156 (38)	1‡	
Other SSTIs	161 (30)	22 (19)	139 (34)		

Abbreviations: MRSA, methicillin-resistant *Staphylococcus aureus*; OR, odds ratio; CI, confidence interval; SSTIs,

skin and soft-tissue infections.

* Adjusted for erythema ≥10 cm and diagnosis group.

†The trunk was defined as the body from and including the axillae to the upper one third of the thighs.

‡For modeling purposes, cellulitis and other SSTIs were grouped.

**Table 5:** Proportion of MRSA by erythema size (N = 500)

Group	MRSA, No./0-4 cm, No.(%)	MRSA, No./5-9 cm, No.(%)	MRSA, No./≥10 cm, No.(%)
All SSTIs (N = 500)	52/309 (17)	20/93 (22)	38/98 (39)
Abscess (N = 178)	23/91 (25)	15/43 (35)	26/44 (59)
Cellulitis (N = 165)	11/81 (14)	3/39 (8)	11/45 (24)
Other SSTIs (N = 157)	18/137 (13)	2/11 (18)	1/9 (11)

Abbreviations: MRSA, methicillin-resistant *Staphylococcus aureus*; SSTIs, skin and soft-tissue infections.

Follow-up was available for 399 (75 percent) of 529 SSTIs, 383 (96 percent) of which improved or resolved within seven days of initial visit, with no difference between MRSA and non-MRSA SSTIs (p = 0.60). Patients lost to follow-up were less likely to have erythema ≥10 cm (p = 0.004).

Initial treatment of the 185 abscesses consisted of antibiotic therapy in 172 (93 percent), I&D in 154 (83 percent), both antibiotic therapy and I&D in 142 (77 percent), and neither in one (1 percent). Empirical antibiotic coverage was common for abscesses at all levels of erythema size: 79 of 91 (87 percent) with 0-4 cm erythema, 42 of 43 (98 percent) with 5-9 cm erythema, and 44 of 44 (100 percent) with ≥10 cm erythema. Beta-lactam antibiotics, TMP/SMX, and combinations of beta-lactam antibiotics with drugs typically active against CA-MRSA were the most frequent choices (Table 6). A minority of abscesses (26 [14 percent]) were treated with outpatient intravenous antibiotic infusion, most often (24 of 26) at the initial visit. Only two patients were hospitalized, one at the initial visit, and one a week after the initial visit, the latter for a drug reaction.

**Table 6:** Initial antibiotic selections for abscesses (N = 172)

Antibiotic selection	Abscesses, No. (%)
Beta-lactam	68 (40)
Cephalosporin	54 (31)
Amoxicillin-clavulanic acid	9 (5)
Amoxicillin	1 (1)
Dicloxacillin	1 (1)
Combination beta-lactam	3 (2)
MRSA-active*	63 (37)
TMP/SMX	45 (26)
Vancomycin	7 (4)
Clindamycin	4 (2)
Tetracycline	1 (1)
Combination MRSA-active	6 (3)
Beta-lactam plus MRSA-active	33 (19)
Fluoroquinolone	8 (5)

Abbreviations: TMP/SMX, Trimethoprim-sulfamethoxazole; MRSA, methicillin-resistant *Staphylococcus aureus*

* Drugs typically active against MRSA by susceptibility testing at PAMF-PA.

Of 152 abscesses with follow-up information, 149 (98 percent) improved or resolved within seven days, 19 (13 percent) required repeat I&D, and 57 (38 percent) had ≥3 follow-up visits. The initial antibiotic selection was active against the isolate in 110 of 125 (88 percent) abscesses with susceptibility results. However, an active drug regimen did not lower the risk for repeat I&D or for ≥3 follow-up visits (p = 0.09 and p = 0.27 respectively). Resource utilization varied by erythema size and was highest in abscesses with erythema ≥10 cm (Table 7). Abscesses caused by MRSA were more likely to require utilization of any resource (31 of 60 [52 percent]) than non-MRSA abscesses (30 of 92 [33 percent]) (p = 0.02).

**Table 7:** Resource* utilization for abscesses by erythema size (N = 143)^†^

Resource	0-4 cm erythema (N = 63)	5-9 cm erythema (N = 41)	≥10 cm erythema (N = 39)
Outpatient IV	3 (5)	3 (7)	18 (46)
Hospitalization	0 (0)	1 (2)	1 (3)
Repeat I&D	5 (8)	5 (12)	8(21)
≥3 Follow-Up visits	11 (18)	15 (37)	28 (72)
Any resource	14 (22)	14 (34)	30 (77)

Abbreviations: IV, Intravenous infusion; I&D, incision and drainage.

*Resources in addition to initial I&D and/or oral antibiotic treatment.

^†^Abscesses with follow-up information and erythema measurement.

## Discussion

In this study of purulent or fluid-producing SSTIs, the prevalence of MRSA was 22 percent. This is substantially below the 42 to 64 percent range previously reported in the US [1-7]. While MRSA overall was less common in our patients, its proportion varied by risk factors. For example, when both abscess diagnosis and large erythema were present, MRSA was isolated in almost 60 percent of cases. Other risk factors for MRSA included a history of prior MRSA in the patient or a close contact, and age <65 years. A history of prior MRSA in the patient had a positive predictive value for current MRSA of 88 percent. Most abscesses were treated empirically with antibiotics, although selection of a drug to which the isolate was susceptible did not affect the outcomes we measured. Seventeen percent of abscesses did not undergo I&D at the initial visit, and 13 percent required a repeat I&D. Resource utilization for abscesses was highest for those with large erythema.

There are several possible explanations for the relatively low prevalence of MRSA in our patients. First, MRSA may have simply emerged more slowly in our region. Indeed, the prevalence of MRSA has been shown to vary geographically [6,25]. However, a surge of MRSA in SSTIs in the Bay Area [1,2] preceding our study makes this unlikely. Second, demographical differences may have contributed to a disparity in MRSA prevalence. Our patients resided in suburban zip codes with relatively high SES, and most had established primary care. In contrast, patients in earlier studies were identified in hospital-based clinics and emergency departments of urban medical centers that serve the poor [1-7]. Urban areas with the lowest income levels have been shown to have the highest incidence rates of MRSA [32], possibly related to clustering of MRSA cases in public housing and incarceration [15]. Third, the distribution of SSTIs into abscesses, cellulitis, and other SSTIs was almost equal in our study. By comparison, abscesses predominated in several previous studies [1,2,6]. The lower proportion of abscesses in our study could have decreased the prevalence of MRSA as we found MRSA to be more common in abscesses. Finally, SSTIs in our study were not limited to those caused by *S. aureus *[4,5]. Limiting our analysis to *S. aureus *alone would have resulted in a MRSA prevalence of 42 percent.

Our findings have implications for the treatment of SSTIs in primary care. For example, clinicians should consider diagnosis type when making antibiotic selections. We found that most culturable, non-abscess SSTIs are caused by bacteria other than MRSA. In fact, in the other SSTIs diagnosis group the proportion of MRSA was only 8 percent if cases of folliculitis, a potential precursor to abscess formation, were excluded. MRSA also appears to be uncommon in non-culturable cellulitis which is usually caused by Streptococcal species [33,34] and responds to beta-lactam antibiotics [34]. In contrast, we found MRSA to be common in abscesses with large erythema. Thus, antibiotics chosen for these infections should be MRSA-active based on local susceptibility testing. However, the imperative for antibiotic treatment of abscesses remains unclear [35-37]. Two randomized, controlled trials (RCTs) of TMP/SMX versus placebo for the treatment of skin abscesses after I&D showed no benefit of drug therapy in reducing treatment failure [38,39]. From this standpoint, the high level of antibiotic coverage for abscesses observed in our study (93 percent) seems excessive. However, in both RCTs there were fewer new lesions in the TMP/SMX group after follow-up, leaving open the possibility of a drug benefit. Also, most abscesses in these studies had erythema or induration of <10 cm. Thus, the validity of their results for larger abscesses is unclear. One observational study of 531 community-acquired SSTIs (mostly abscesses) caused by MRSA that excluded "minor or superficial skin infections" found that lack of an active antibiotic was a risk factor for treatment failure [19]. While we did not find an effect of active antibiotic therapy on the frequency of repeat I&D or follow-up visits for abscesses, the generalizability of our results is limited by the small sample size and observational design of our study.

In addition, some I&D procedures may be delayed or inadequate. While we are not aware of validated criteria by which to make these judgments, it is reasonable for clinicians to consider aspiration or ultrasound imaging if the presence of pus is unclear, or surgical consultation if there is doubt about the examiner's ability to perform an I&D.

Finally, greater resources are expended on abscesses with large erythema. Why some abscesses are large at presentation is unknown. Possible modifiable factors include delay in seeking medical care, attempts at self-treatment (e.g. squeezing or even incising the abscess), delayed or inadequate I&D, and inappropriate antibiotic selection or route of antibiotic delivery.

Our study also has implications for the prevention of SSTIs caused by MRSA. The strong association of prior MRSA in the patient or a close contact with current MRSA suggests that persistent MRSA colonization in the individual [40], and transmission of MRSA in households [41] contribute to incident MRSA SSTIs. The smaller proportion of SSTIs caused by MRSA among patients ≥65 years may reflect lower potential exposure through skin-to-skin contact or contaminated fomites as the elderly more typically live alone [42]. De-colonization and limiting fomite exposure among household contacts are two possible strategies for limiting the spread of MRSA in the community.

### Limitations

There are several limitations to our study. First, only SSTIs that produced pus or fluid were included in the study. Excluding non-culturable cellulitis, which is largely Streptococcal in origin [33,34], may have biased our estimation of the prevalence of MRSA upward. Second, the decision to culture an SSTI was left to the discretion of the provider. We do not know how many culturable SSTIs were not cultured. However, the observation that 68 percent of SSTIs undergoing I&D in urgent care during the study period were included in the study suggests case identification was good. Third, 25 percent of our patients were lost to follow-up and our follow-up extended to only one week. We may have missed some treatment failures or late complications. Finally, the small sample size and lack of experimental design of our study limited our ability to detect a treatment effect of active antibiotic therapy on outcomes for abscesses.

Future studies are needed to confirm the high proportion of MRSA in large abscesses, to examine the effect of adjunctive antibiotics in the treatment of abscesses (especially large abscesses), to define quality measures for I&D, and to determine why some SSTIs are large at presentation.

## Conclusions

The prevalence of MRSA is relatively low in a broad range of SSTIs diagnosed in suburban primary care. In contrast, MRSA is the most common isolate in abscesses with large erythema. Empirical antibiotic treatment, if selected for these infections, should be active against MRSA. Many non-abscess SSTIs do not require empirical coverage for MRSA and over-treatment of small abscesses with antibiotics appears to be common.

Documentation of prior MRSA in health records may alert clinicians to the high probability of MRSA in subsequent culturable SSTIs.

## Competing interests

All authors declare no competing interests.

## Authors' contributions

CW, PK, and JB conceived and designed the study. CW wrote the first draft of the paper PK and JB contributed to the final draft. CW and PK were responsible for conducting the study and managing the data. CW, PK, and CL conducted the statistical analysis and interpretation of the data. All authors read and approved the final manuscript.
